# The Genesis of Epidemic Diseases

**Published:** 1907-03-09

**Authors:** 


### The Hospital

A Journal of

The Medical Sciences and Hospital Administration.

Vol. XLI.—No. 1069. SATURDAY, MARCH 9, 1907.

### The Genesis of Epidemic Diseases.

Among the many problems which lie awaiting solution at the hands of the youthful and energetic science of bacteriology is the important question which concerns the origin of large epidemics. Why does Asiatic cholera, to take the most striking instance, at times manifest a tendency to spread suddenly from its habitat in India and the East and to sweep over countries where it has hitherto been a stranger? There must be reasons, we may be sure, underlying this tremendous access of vitality on the part of the cholera bacillus. The history of influenza, in like manner, shows that here also is a disease which, for some unknown reason, becomes epidemic, and, indeed, pandemic, in a series of waves, retiring as mysteriously as it appears, to some unknown endemic area where the germ is, so to speak, native. To take another instance, Why has plague, during the last ten or fifteen years, kept knocking so importunately at the door of Western Europe?

It is always easier to propound problems than to solve them, and yet, it seems to us that this particular problem might be more easily solved than might at first sight appear. True, the amount of information at our disposal bearing directly on the question is very scanty, but suggestive knowledge, drawn by analogy from regions of vital activity other than microbic, may indirectly throw some light on the matter, and indicate to workers the lines upon which to travel to reach the end desired. Arguing strictly *a priori*, one would be inclined to say that there can only be two causes which can lead to the extension of the habitat of a species, whether that species be animal or vegetable. These causes are an increase in the vitality and strength of the organism, which enables it to cope with conditions previously fatal to it, or an alteration of these conditions in such a manner as to render them either harmless or actually beneficial to the organism. It is well known, for instance, that some tropical diseases flourish only within certain well-defined areas, and that their excursions from these areas are conditioned by such simple alterations in environment as a rise of the mean temperature of a locality. If, therefore, the general mean temperature of the globe were elevated, we would doubtless experience an extension in the range of many diseases. But alterations of atmospheric and thermal conditions are rare, and the more probable explanation of these sudden and far-travelling epidemics lies rather in the first possibility—namely, in the circumstance that the organism passing through a series of favourable conditions is thereby rendered more vigorous, more hardy, and better fitted to defy factors which are, generally speaking, too severe for its natural or normal delicacy to overcome. We may, therefore, conclude that the causes favouring a sudden migration of a disease from its normal habitat to others lie not so much in an alteration of external conditions as in a change in the powers of resistance possessed by the organism itself. There is evidence that this may occur in those microbes which produce the great plagues of mankind. It is a well-known laboratory fact that the virulence of certain organisms can be increased or diminished at will, as a rule, by altering the culture medium in which they are grown. In other words, the more favourable conditions not only favour the bacterial growth by providing a more suitable environment, but also actually increase the vigour of the organism. It is also known that this acquired virulence lasts through several generations.

Leaving the artificial surroundings of the laboratory and coming to the outer world, we again meet with several highly suggestive facts. Every practising physician knows that epidemics vary in severity, and that the most fatal epidemics are at the same time the most widespread. Again, it is said that within the last fifty years physicians no longer see such terrible individual cases of small-pox and of syphilis as they did in earlier days. The reason adduced for the present-day mildness of these diseases is that the organisms responsible for them have become profoundly modified, in the one case through the influence of compulsory vaccination having attenuated the virus of small-pox by compelling it to pass through individuals protected against it, and in the other, syphilis, by the general treatment of most cases by mercury. In both these epidemic diseases an environment inimical to the germ has induced an essential weakening of the strain.

Here are examples of the microbe having been weakened by passage through a powerfully resisting human pabulum. Like a see-saw, when the one side is up, the other side is down. What more probable than that the contrary is also true? Where a large community is depressed in its general health and resisting power, a virulent strain of microbes will be generated and begin its conquering march round the world by virtue of its own innate super-vitality. If this be true, then the spread of a disease like cholera, for example, implies a preliminary deterioration in the health of the masses in India where the disease has its natural dwelling-place. Thus the presence at one part of the globe of famine, vice, and their dread sister, pestilence, is fraught with peril not only to the country immediately concerned, but to the human race as a whole. The conclusion is one that "gives furiously to think."

